# Interference KRT17 reverses doxorubicin resistance in triple-negative breast cancer cells by Wnt/β-catenin signaling pathway

**DOI:** 10.1007/s13258-023-01437-y

**Published:** 2023-08-27

**Authors:** Liqiong Wu, Wenshuang Ding, Xiaopai Wang, Xiubo Li, Jing Yang

**Affiliations:** 1 Department of Pathology, Guangzhou First People’s Hospital, 51080 Guangzhou, R.P. China; 2 The Second Affiliated Hospital of South, China University of Technology, 51080 Guangzhou, R.P. China

**Keywords:** KRT17, Doxorubicin resistance, Triple-negative breast cancer, Wnt/β-catenin signaling pathway

## Abstract

**Background:**

Triple-negative breast cancer (TNBC) is a subtype of breast cancer with the highest degree of malignancy and is easily resistant to drugs due to the lack of hormone receptors. Research on the resistance mechanisms in TNBC is particularly important. Keratin 17 (KRT17) is highly expressed in TNBC. Anthracycline doxorubicin (Dox) is a commonly used chemotherapeutic drug for early stage triple-negative breast cancer.

**Objective:**

This study investigated the role of KRT17 in TNBC-Dox resistance.

**Methods:**

Immuno-histochemical staining, qPCR, western blotting (WB), and immunofluorescence were used to detect the expression of KRT17 in TNBC-Dox-resistant patients and in TNBC-Dox-resistant MDA-MB-468 and MDA-MB-231. the effect of KRT17 on the proliferation and migration in KRT17 knockdown of TNBC-Dox-resistant cells was determined by the CCK8, clone formation, transwell invasion and wound healing assays were used to determine.

**Results:**

KRT17 was highly expressed in the TNBC-Dox-resistant cells. Knockdown of KRT17 significantly reduced the IC50s of TNBC-Dox-resistant and parental strains and also reduced the proliferation and invasion abilities of TNBC-Dox-resistant cell lines. KRT17 regulated the Wnt/β-catenin signaling pathway. The inhibitory effect of KRT17 knockdown on the proliferation and migration of TNBC-Dox-resistant cells was reversed by an activator of the Wnt signaling pathway.

**Conclusion:**

KRT17 can inhibit the Wnt/β-catenin signaling pathway, thereby reducing the proliferation and invasion ability of TNBC-Dox-resistant cells.

## Introduction

Triple-negative breast cancer (TNBC) is a specific subtype of breast cancer that lacks the estrogen receptor (ER) (Depianto et al. 2010), progesterone receptor (PR) (Dey et al. 2013), and the human epidermal growth factor receptor 2 (HER-2). It is highly aggressive, has strong metastatic ability, poor prognosis, and other clinical features (Yin et al. 2020). TNBC is resistant to hormone and immune-targeted therapy owing to a lack of hormone and growth factor receptors. Doxorubicin (Dox) is a chemotherapeutic drug commonly used for treating early breast cancer. It is an anthracycline antibiotic with broad-spectrum anti-tumor effects, primarily used as a topoisomerase inhibitor. It binds to the DNA replication fork, inhibits DNA replication, and induces apoptosis and autophagy (Delgado et al. 2018; Nitiss 2009; Rhee et al. 2007). However, patients are prone to developing resistance to the chemotherapeutic drugs, which is one of the main causes of chemotherapy failure. Therefore, it is crucial to investigate the mechanism of doxorubicin resistance in breast cancer and to identify target genes that inhibit drug resistance.

Keratin 17 (KRT17) is a type I keratin that is involved in multiple biological processes such as tumor growth and metastasis, inflammation, and signal transduction (Depianto et al. 2010; Yang et al. 2019). KRT17 is highly expressed in cervical, pancreatic, gastric, lung, and other cancers and is involved in the resistance mechanisms of several chemotherapeutic drugs (Li et al. 2021b). KRT17 knockdown inhibited proliferation and migration of gastric cancer cells (Chivu-Economescu et al. 2017) and also significantly inhibited paclitaxel-induced drug resistance in cervical cancer cells (Li et al. 2019). The expression rate of KRT17 in triple-negative breast cancer exceeds 80%, whereas the expression rate in non-TNBC is less than 50%, and its expression is positively correlated with poor breast cancer prognosis (Merkin et al. 2017).

The Wnt/β-catenin signaling pathway is a classic Wnt signaling pathway involved in a series of physiological processes, such as embryonic development, stem cell homeostasis, cancer occurrence and development, and tumor drug resistance. This pathway is mainly activated through the binding of Wnt ligands to Frizzled (FZD) and LRP5/6 membrane receptors, recruiting scaffold proteins such as Axis Inhibition Protein (AXIN) and Dishevelled Segment Polarity Protein (DVL). The Wnt/β-catenin signaling pathway is also regulated by the Wnt pathway regulator, Adenomatous Polyposis Coli protein (APC). β-catenin is released, enters the nucleus and is activated by binding to the Transcription Factor (TF). Transcription factors initiate epithelial-mesenchymal transition (EMT), growth, and transduction-related gene expression (Azzolin et al. 2014; Clevers and Nusse 2012; Yang et al. 2016). Studies have shown that the Wnt/β-catenin signaling pathway is specifically activated in TNBC and is associated with drug resistance in TNBC (Dey et al. 2013; Merikhian et al. 2021).

This study discussed the role of KRT17 in TNBC-Dox resistance. The effect of KRT17 on the Wnt/β-catenin signaling pathway and the induction of TNBC-Dox resistance, as well as the proliferation and migration of TNBC-Dox-resistant cells were also investigated, thus providing evidence for KRT17 as a TNBC-Dox-resistant therapeutic target.

## Materials and methods

### Cell culture, cell transfection, and construction of stably transfected cell lines

The TNBC cell lines MDA-MB-468 (ATCC, HTB-132) and MDA-MB-231 (ATCC, HTB-26) were purchased from the ATCC (Rockefeller, MD, USA). The 293T cell lines were maintained in our laboratory. The MDA-MB-468, MDA-MB-231, and 293T cell lines were preserved in MEM medium supplemented with 10% FBS. Once the cells reached 60–75% confluency, 50 nM small interfering RNA (siRNA) (genechem, Shanghai, China) of KRT17 was transfected into TNBC cells using Lipofectamine 2000 reagent (ThermoFisher, Waltham, USA) according to the manufacturer’s instructions. The cells were harvested after 48 h incubation. The siRNA sequences were as follows: 5’-GCCAACAUCCUGCUACAGAUU-3’, 5’-GCGUGACCAGUAUGAGAAGAU-3’, and 5’-CACCUGACUCAGUACAAGAAA-3’. Stable transfected cell lines were constructed by sub-cloning the most effective siRNAs, siRNA2 and si-NC, into a lentivirus vector (pLKO.1) (addgene, Beijing, China) resulting in the construction of sh-KRT17 and sh-NC vectors. The shKRT17 and sh-NC vectors were transfected into 293T cells for lentivirus preparation. The viruses were harvested to infect MDA-MB-468 and MDA-MB-231 cells, followed by selection with puromycin.

### RT-qPCR

According to the manufacturer’s instructions, the total RNA of MDA-MB-468 and MDA-MB-231 cells was extracted using Trizol reagent and reverse-transcribed into cDNA using the PrimeScript RT kit (Takara Bio, Osaka, Japan). The SYBR® Premix Ex Taq™ II kit (Takara Bio, Osaka, Japan) was used for RT-qPCR. The RT-qPCR cycling conditions were: 95℃ for 10 min, 55℃ for 2 min, 72℃ for 2 min, followed by 40 cycles of 95℃ for 15 s and 60℃ for 1 min. The primers used for RT-qPCR analysis were as follows: KRT17 forward primer, 5′-ATCCTGCTGGATGTGAAGACGC-3′ and KRT17 reverse primer, 5′-TCCACAATGGTACGCACCTGAC-3′; GAPDH forward primer, 5′-GTCTCCTCTGACTTCAACAGCG-3’; and GAPDH reverse primer, 5′-ACCACCCTGTTGCTGTAGCCAA-3′. The 2^−ΔΔCq^ method was used to calculate the KRT17 expression levels.

### Western blotting

The MDA-MB-468 and MDA-MB-231 cells were lysed according to the manufacturer’s instructions. The protein levels were measured using the BCA protein assay kit. Equal amounts of denatured proteins were transferred to a polyvinylidene fluoride membrane (Millipore, Billerica, USA) and incubated (4℃, overnight) with primary antibodies KRT17 (1:500, ab53707, Abcam, Cambridge, England), β-catenin (1:1 000, 9562, CST, Danvers, USA), APC (1:500, ab40778, Abcam, Cambridge, England), AXIN1 (1:1000, 2087, CST, Danvers, USA), GAPDH (1:5000, 10494-1-AP, Proteintech, Wuhan, China). The membrane was then incubated with goat anti-rabbit secondary antibody (1:10 000, SA00001-2, Proteintech, Wuhan, China). The blots were visualized using an enhanced chemiluminescence reagent ( ThermoFisher, Waltham, USA).

### Fluorescence in situ hybridization (FISH)

KRT17 (1 µg/mL, ab53707, Abcam, Cambridge, England) was used to observe the localization of KRT17 in MDA-MB-468 and MDA-MB-231 cells. The Fluorescent In Situ Hybridization Kit (GenePharma, Shanghai, China) was used to perform the FISH assay, according to the manufacturer’s instructions. The cell nuclei were stained with 4,6-diamidino-2-phenylindole (DAPI; Beyotime, Shanghai, China). Images were obtained using a fluorescence microscope (Leica, Wetzlar, Germany).

### CCK8 assay

A total of 2.5 × 10^3^ cells/well of MDA-MB-468 and MDA-MB-231 were added to a 96-well plate and incubated for 60 min. Ten microliters of CCK8 reagent was added at 0, 24, 48, and 72 h. Optical density was measured at 450 nm using an enzyme-labeled instrument (Thermo Fisher, Waltham, USA).

### Transwell invasion assays

MDA-MB-468 and MDA-MB-231 cells (1 × 10^5^) were placed in the top chamber of the 24-well Transwell plate coated with Matrigel containing 200 µL serum-free medium. The bottom chamber contained complete medium supplemented with 10% FBS. After 48 h, cells were fixed with anhydrous ethanol for 20 min and stained with crystal violet for 15 min. Images were obtained under an inverted microscope (Leica, Wetzlar, Germany).

### Wound healing

Approximately 1 × 10^6^ cells were added to a six-well plate. The following day, a pipette tip was used to scratch parallel lines on the cell layer. The cells were then washed three times with phosphate-buffered saline, the streaked cells were removed, and serum-free medium was added. The remaining cells were then placed in a 37 °C, 5% CO_2_ incubator and cultivated. Images were acquired after 0 and 24 h.

### Statistical analysis

Data were expressed as mean ± standard deviation (SD). One-way analysis of variance (ANOVA) and Dunnett’s post-hoc test were performed on more than two groups of data for statistical analysis, and the Student’s t-test was used to analyze the means of the two groups. Statistical significance was set at *P* < 0.05. All experiments were repeated in triplicate.

## Results

### KRT17 is highly expressed in TNBC-Dox-resistant cells

**Fig. 1:**
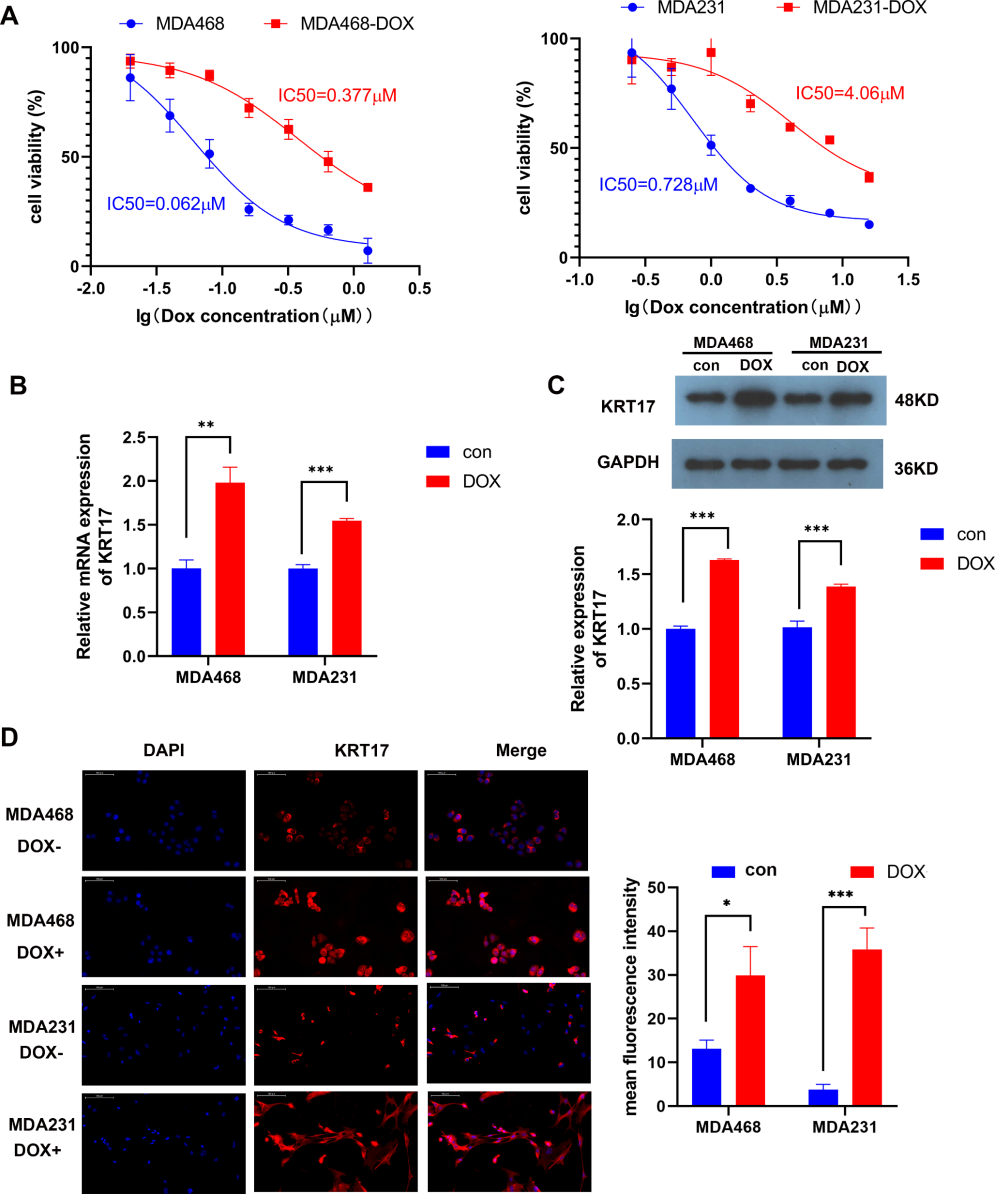
KRT17 is highly expressed in TNBC-Dox-resistant cells. (**A**) Constructed in MDA-MB-468-DOX and MDA-MB-231-DOX resistant cells respectively and tested for parental resistance to Dox resistant cells by assaying IC50. (**B**) qPCR detection of KRT17 expression in MDA-MB-468 and MDA-MB-231 parental and Dox-resistant cells. (**C**) KRT17 protein level detected by WB in MDA-MB-468 and MDA-MB-231 parental and Dox-resistant cells. (**D**) Immunofluorescence detection of KRT17 expression and cellular localization in MDA-MB-468 and MDA-MB-231 parental and Dox-resistant cells

TNBC patients are resistant to endocrine and hormone therapies due to the lack of endocrine and hormone receptors, however tumor growth and metastasis are mainly controlled by chemotherapy. Dox is a common chemotherapeutic drug used to treat breast cancer. However, after multiple chemotherapy treatments, patients develop resistance to Dox. Previous studies have shown that KRT17 is highly expressed in TNBC tissues (Merkin et al. 2017). KRT17 may be involved in the mechanism of Dox resistance in TNBC, thus Dox-resistant strains were constructed in the TNBC cell lines, MDA-MB-468 and MDA-MB-231, and their IC50s were determined. The IC50s of the Dox-resistant strains were significantly higher than those of the parental strains (Fig. 1A). Western blotting, qPCR, and immunofluorescence showed that KRT17 was highly expressed in TNBC-Dox-resistant cells compared with the parental strains (Fig. 1B and D).

### KRT17 knockdown inhibits dox resistance of TNBC cells

**Fig. 2:**
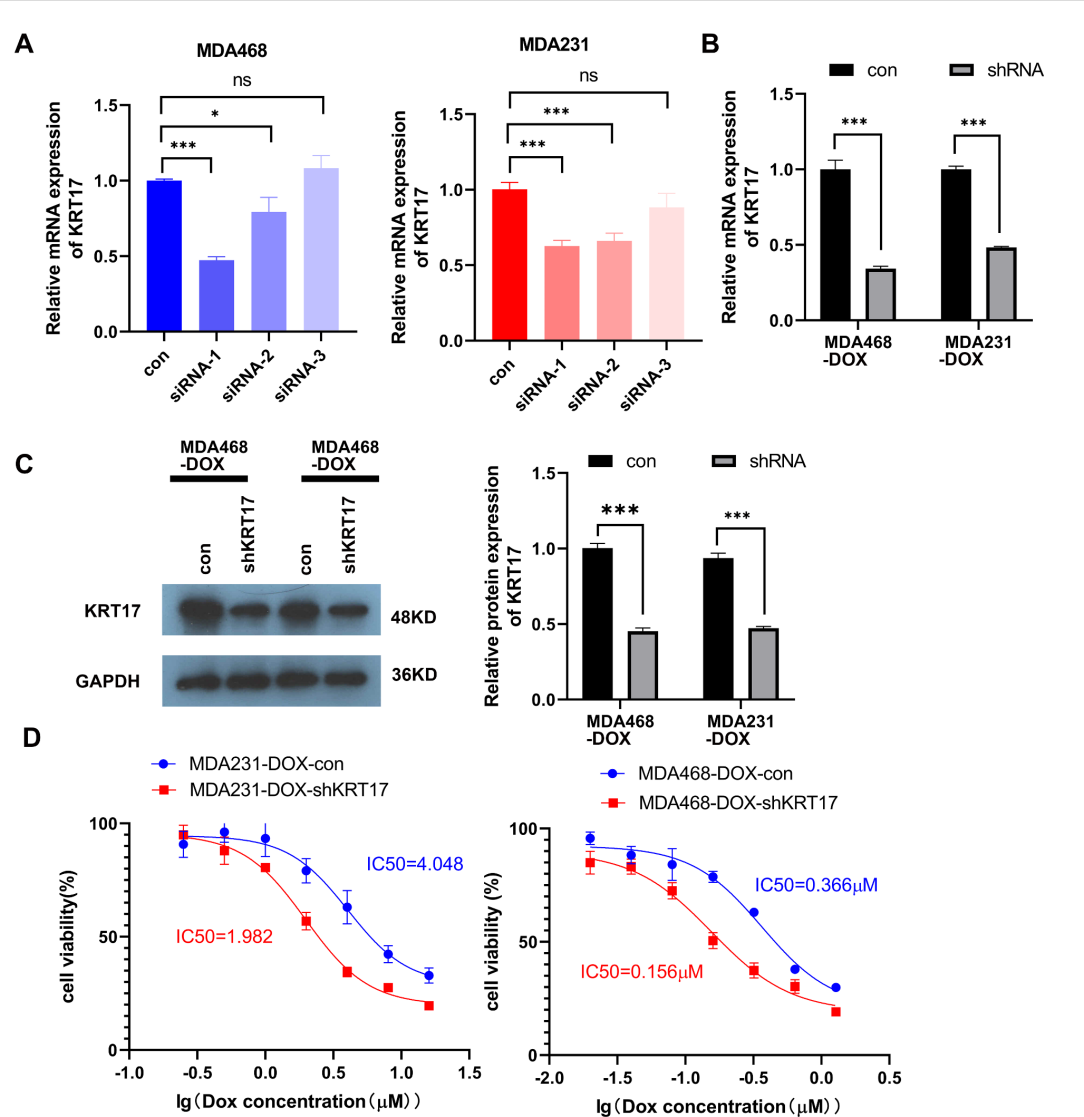
KRT17 knockdown inhibits Dox resistance in TNBC cells. (**A**) qPCR detection of siRNA infection efficiency in MDA-MB-468-Dox and MDA-MB-231-Dox-resistant cells. (**B, C**) qPCR and WB detection of KRT17 knockdown in TNBC-Dox-resistant cells KRT17 stable cell line. (**D**) IC50s were detected in TNBC-Dox-resistant knockdown KRT17 stable cell lines

The effect of KRT17 on TNBC-Dox resistance was determined by using siRNA to knockdown KRT17 expression in TNBC-Dox-resistant strains. Three siRNAs were also constructed to transfect MDA-MB-468-Dox and MDA-MB-231-Dox. The efficiency of knockdown siRNA was detected by qPCR (Fig. 2A). The siRNA-1 construct exhibited the best knockdown effect, and was selected to construct a stable KRT17 knockdown cell line in TNBC-Dox-resistant cell lines. The results of qPCR and WB showed that the expression of KRT17 was significantly downregulated in the TNBC-Dox-resistant cell lines (Fig. 2B C). In Dox-resistant strains, KRT17 knockdown significantly reduced the IC50s of the cells (Fig. 2D). Therefore, KRT17 knockdown increased the sensitivity of TNBC cells to Dox.

### KRT17 knockdown inhibits the proliferation and migration of TNBC-Dox-resistant strains

**Fig. 3:**
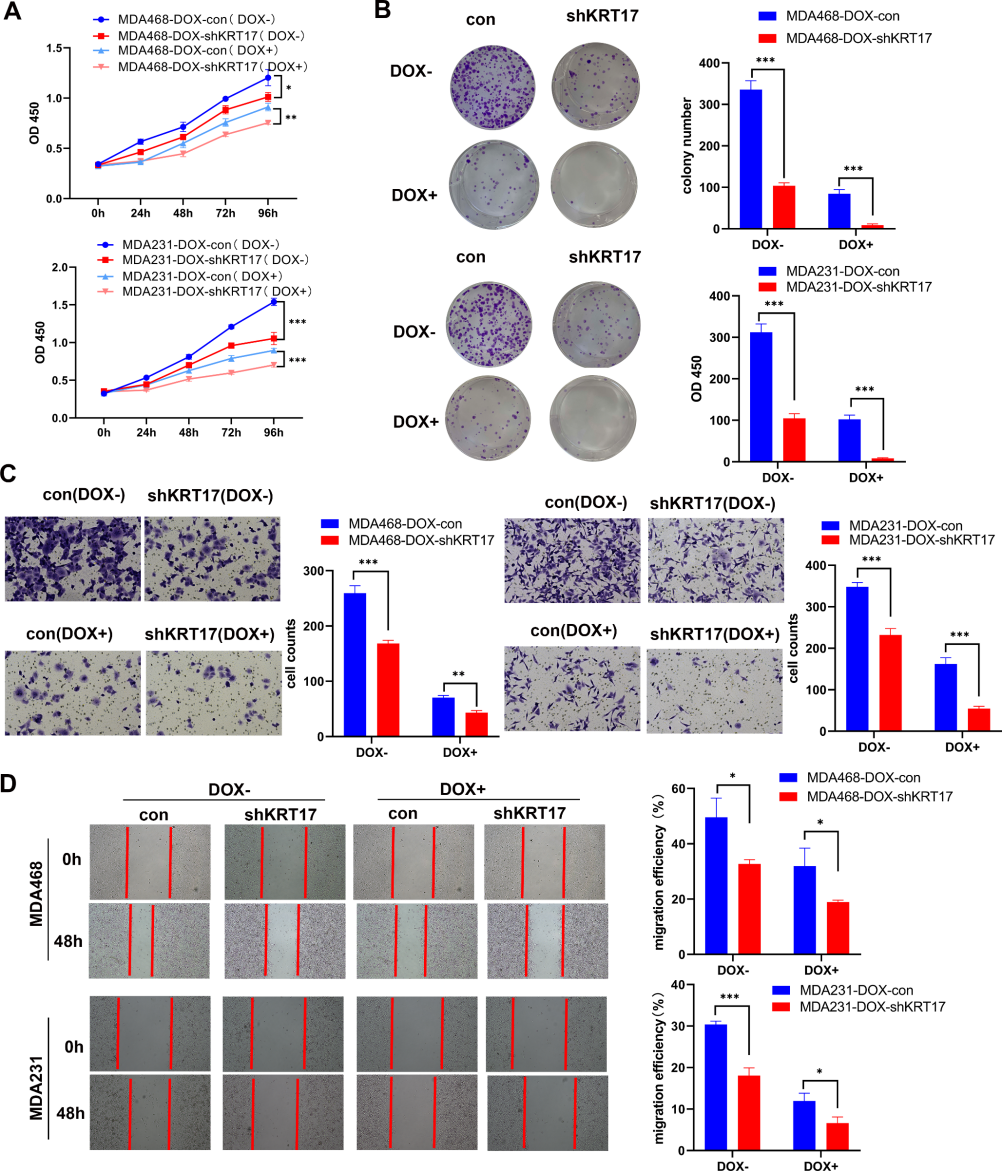
KRT17 knockdown inhibited the proliferation and migration of TNBC-Dox-resistant strains. (**A**) The effect of KRT17 knockdown on the proliferation of TNBC-Dox-resistant strains was detected by CCK8, with and without Dox. (**B**) The clone formation assay detected the monoclonal formation ability of KRT17 knockdown against TNBC-Dox-resistant strains, with and without Dox. (**C**) Transwell invasion assays used to detect the effect of knockdown of KRT17 on the invasion ability of TNBC-Dox-resistant strains, with and without Dox. (**D**) The effect of KRT17 knockdown on the growth and migration of TNBC-Dox-resistant strains was examined by the wound healing assay

The effect of KRT17 on the proliferation and migration of TNBC-Dox-resistant strains was clarified by examining cell function with and without Dox (MDA468-DOX: 0.4 µM, MDA231-DOX: 4 µM) addition in normal and KRT17-knockdown TNBC-Dox-resistant strains. The CCK8 and clone formation demonstrated that KRT17 knockdown significantly reduced the proliferative ability of TNBC-resistant strains in the presence and absence of Dox (Fig. 3A and B). Transwell and wound healing assays showed that KRT17 knockdown significantly inhibited the invasion ability of TNBC-resistant strains, with and without Dox addition (Fig. 3C and D). Thus, KRT17 knockdown inhibited the proliferation and invasion abilities of TNBC-Dox-resistant strains, with or without addition of Dox.

### KRT17 knockdown inhibits the Wnt/β-catenin signaling pathway

**Fig. 4:**
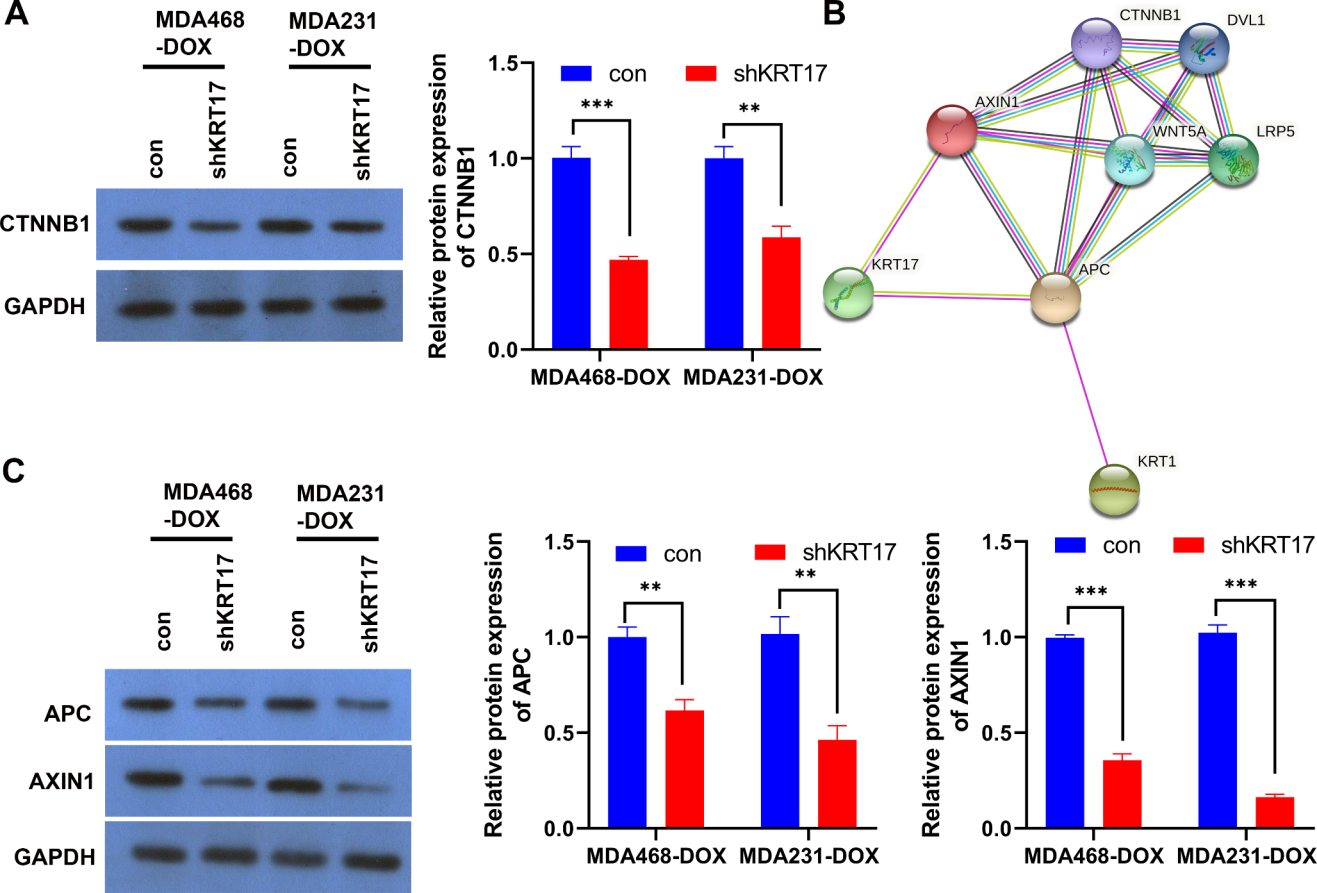
KRT17 knockdown inhibits the Wnt/β-catenin signaling pathway. (**A**) WB detection of the effect of KRT17 knockdown on β-catenin expression in TNBC-Dox-resistant cells. (**B**) STRING database analysis of the regulatory network of KRT17 and Wnt/β-catenin signaling pathway. (**C**) The effect of KRT17 on the expression of APC and AXIN protein was detected by WB.

Aberrant Wnt signaling is characteristic of triple negative breast cancer, compared to other subtypes of breast cancer. Numerous studies have shown that TNBC is associated with the activation of the Wnt/β-catenin signaling pathway, the activation of which increases the metastasis and poor prognosis of breast cancer (Geyer et al. 2011; Khramtsov et al. 2010). Therefore, the activation of the Wnt/β-catenin signaling pathway in the KRT17 knockdown TNBC-Dox-resistant strains was determined. Western blotting showed that KRT17 knockdown inhibited the activation of the Wnt/β-catenin signaling pathway (Fig. 4A). The STRING database was used to predict the regulatory network of KRT17 and Wnt/β-catenin signaling pathways (Fig. 4B). The results showed that KRT17 and the Wnt/β-catenin signaling pathways are negative regulators of APC and AXIN1 and may mutually regulate each other. The WB results showed that KRT17 knockdown increased the expression of APC and AXIN1, especially of AXIN1 (Fig. 4C).

### Wnt activator reverses the inhibitory effect of interfering KRT17 on the proliferation and migration of TNBC-Dox-resistant strains

**Fig. 5:**
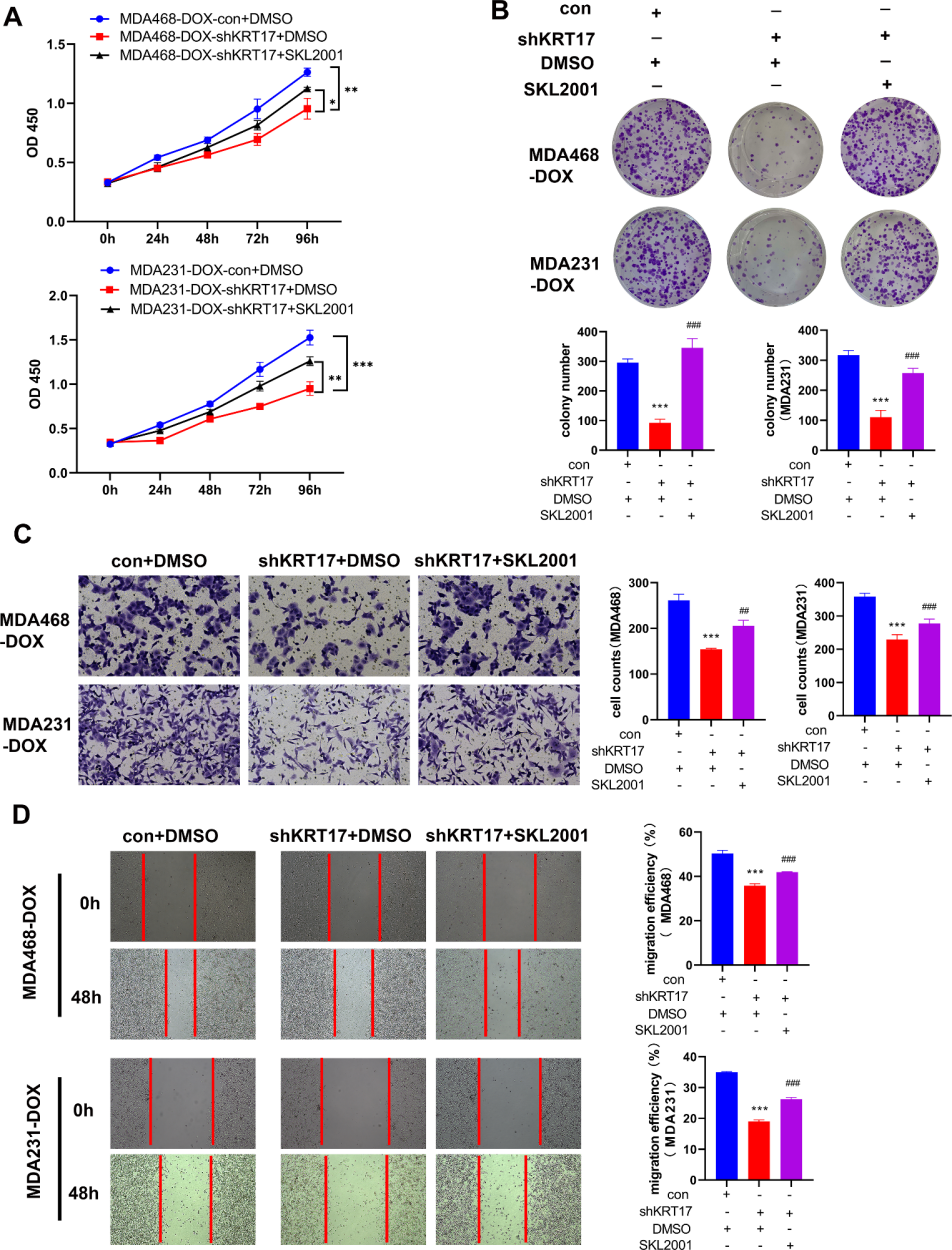
Wnt activator reverses the inhibitory effect of interfering KRT17 on proliferation and migration of TNBC-Dox-resistant strains. (**A**) The reversal effect of Wnt/β-catenin activator on the proliferation inhibition of TNBC-Dox-resistant strains by KRT17 knockdown was detected by CCK8. (**B**) Clone formation assay to detect the reversal effect of Wnt/β-catenin activator on the inhibition of monoclonal formation of TNBC-Dox-resistant strains by knockdown of KRT17. (**C**) Transwell invasion assays were used to detect the reversal effect of Wnt/β-catenin activator on KRT17 knockdown on the invasive ability of TNBC-Dox resistant strains. (**D**) Wound healing was used to detect the effect of Wnt/β-catenin activator on KRT17 knockdown on TNBC-Dox resistance reversal of inhibition of growth and migration ability of drug strains

Since interfering with KRT17 expression can activate the Wnt/β-catenin signaling pathway, the 20 µM Wnt/β-catenin activator SKL2001 was added to KRT17 knockdown TNBC-Dox-resistant cells. SKL2001 disrupts the interaction of Axin/β-catenin by activating the Wnt/β-catenin signaling pathway. SKL2001 was added to determine whether the Wnt/β-catenin activator can reverse the inhibitory effect of interfering KRT17 on the proliferation and invasion of TNBC-Dox-resistant strains. The results of CCK8 and clone formation showed that the Wnt/β-catenin activator could reverse the inhibition of proliferation of TNBC-Dox-resistant strains (Fig. 5A and B). Transwell and wound healing assays showed that the Wnt/β-catenin activator could reverse the inhibition of invasion of TNBC-Dox-resistant strains (Fig. 5C and D).

## Discussion

Owing to the lack of endocrine and anti-HER2 targets for TNBC, most patients are mainly treated with chemotherapy. Dox is a chemotherapeutic drug commonly used in the treatment of early breast cancer. Treatment efficacy is reasonable in the early stages of cancer; however, it is prone to drug resistance in the later stages, and drug resistance is a key factor for tumor metastasis. Merkin et al. found that KRT17 is highly expressed in TNBC and HER2 receptor-negative breast cancer patients (Merkin et al. 2017). Our study showed that KRT17 was highly expressed in TNBC-Dox-resistant cell lines, and knocking down KRT17 enhanced the sensitivity of TNBC and TNBC-Dox-resistant cells to Dox. Furthermore, knocking down KRT17 inhibited the proliferation of TNBC-Dox-resistant cells and its invasive ability. Li et al. also found that interference with KRT17 inhibited the proliferation, migration, and cisplatin resistance of bladder cancer cells (Li et al. 2021a).

The Wnt/β-catenin signaling pathway is a well-established classical signaling pathway that controls embryonic and cancer development. Recently, the Wnt/β-catenin signaling pathway has been shown to be involved in stemness maintenance (Gangrade et al. 2018), drug resistance generation (Piva et al. 2014), proliferation (Butti et al. 2018), metastasis (Lecarpentier et al. 2019), and immune microenvironment regulation (Marra et al. 2019) in breast cancer. Xu et al. found that β-catenin knockdown TNBC cells were more sensitive to the chemotherapeutic drugs, Dox and cisplatin, and β-catenin could control various tumor properties, such as TNBC proliferation and migration (Xu et al. 2015). Our study also showed that the knockdown of KRT17 in TNBC-Dox-resistant cells inhibited the expression of β-catenin, especially, in the nucleus. Furthermore, the Wnt/β-catenin activator SKL2001 was shown to reverse the inhibitory effect of KRT17 knockdown on the proliferation and migration of TNBC-Dox-resistant cells. SKL2001 mainly acts on Axin/β-catenin and releases β-catenin, thereby activating the Wnt/β-catenin signaling pathway.

The relationship between KRT17 and Wnt/β-catenin signaling pathway was predicted using the STRING database. The expression of APC and AXIN1 proteins was increased which is important as APC and AXIN1 proteins are repressors of the Wnt/β-catenin signaling pathway. Ran et al. also found that KRT17 could regulate the Wnt/β-catenin signaling pathway in colon cancer cells by regulating APC (Ji et al. 2021). How KRT17 regulates the expression of APC and AXIN1 proteins will be studied in the future. We hypothesized that that KRT17 directly binds to APC and AXIN1 proteins to promote their ubiquitination and inhibit protein expression. In addition, the effect of KRT17 knockdown on xenografts in animals requires further improvement.

In conclusion, KRT17 knockdown inhibited drug resistance, proliferation, and migration of TNBC-Dox cells by inhibiting the Wnt/β-catenin signaling pathway.

## Data Availability

The datasets generated during and/or analyzed during the current study are available if requested from the corresponding authors.
